# Prevalence and municipal variation in chronic musculoskeletal pain among independent older people: data from the Japan Gerontological Evaluation Study (JAGES)

**DOI:** 10.1186/s12891-022-05694-y

**Published:** 2022-08-05

**Authors:** Keiko Yamada, Tomoko Fujii, Yasuhiko Kubota, Takaaki Ikeda, Masamichi Hanazato, Naoki Kondo, Ko Matsudaira, Katsunori Kondo

**Affiliations:** 1 Department of Anesthesiology and Pain Medicine, Juntendo University Faculty of Medicine, Tokyo, Japan; 2 Pain Medicine, Juntendo University Graduate School of Medicine, Tokyo, Japan; 3 Department of Medical Research and Management for Musculoskeletal Pain, 22nd Century Medical and Research Center, the University of Tokyo Hospital, Tokyo, Japan; 4 Osaka Center for Cancer and Cardiovascular Diseases Prevention, Osaka, Japan; 5 Department of Health Policy Science, Graduate School of Medical Science, Yamagata University, Yamagata, Japan; 6 Department of International and Community Oral Health, Tohoku University Graduate School of Dentistry, Sendai, Japan; 7 Department of Environmental Preventive Medical Sciences, Center for Preventive Medical Sciences, Chiba University, Chiba, Japan; 8 Department of Social Epidemiology, Graduate School of Medicine and School of Public Health, Kyoto University, Kyoto, Japan; 9 Department of Social Preventive Medical Sciences, Center for Preventive Medical Sciences, Chiba University, Chiba, Japan; 10 Department of Gerontological Evaluation, Center for Gerontology and Social Science, National Center for Geriatrics and Gerontology, Aichi, Japan

**Keywords:** Aged, Chronic pain, Cohort studies, Musculoskeletal pain, Widespread chronic pain

## Abstract

**Background:**

Urbanization and population aging may affect prevalence of chronic pain from various causes. This cross-sectional study aimed to investigate the prevalence of chronic musculoskeletal pain, including some subtypes, in independent Japanese older people, and whether population density and population aging rate explained prevalence and differences in pain levels between municipalities.

**Methods:**

We analyzed data from 12,883 independent older people living in 58 municipalities who completed mailed questionnaires and did not need support for daily living. We identified three types of pain: “chronic musculoskeletal pain” lasting ≥ 3 months (overall and in each part of the body), “chronic widespread-type pain” in the spinal and peripheral area, and “chronic multisite pain” in at least three sites. The latter two were measured using new definitions. These types of pain are correlated with depressive symptoms and we therefore examined the construct validity of the definitions by comparing the Geriatric Depression Scale score. We also used analysis of covariance to compare the prevalence of these three types of pain between municipalities. Odds ratios, median odds ratios, and the municipal variance in prevalence of chronic musculoskeletal pain were estimated by Bayesian multilevel logistic regression analysis using the Markov Chain Monte Carlo method.

**Results:**

The construct validity of the definitions of chronic widespread-type pain and chronic multisite pain was confirmed. The prevalence of the three types of pain (chronic musculoskeletal, widespread, and multisite pain) was 39.0%, 13.9%, and 10.3%, respectively. Chronic musculoskeletal pain showed a higher prevalence among older people and women. Individuals in underpopulated, suburban, or metropolitan areas tended to have more pain than those in urban areas, but this was not statistically significant (odds ratio [95% credible interval] 1.15 [0.86–1.51], 1.17 [0.93–1.43], 1.17 [0.94–1.46]). Population density and population aging rate did not explain the differences between municipalities.

**Conclusions:**

The prevalence of chronic musculoskeletal pain was consistent with previous global reports. Areas with overpopulation and depopulation tended to have higher pain prevalence, but population density and population aging rate did not explain municipal variance. Further research is needed to identify other factors that contribute to regional variance.

**Supplementary Information:**

The online version contains supplementary material available at 10.1186/s12891-022-05694-y.

## Background

Population aging may affect the prevalence of chronic pain from various causes associated with injury or illness [1]. A systematic review reported that global prevalence of chronic pain among community-dwelling older people was 25%–76% [1]. However, Japanese data were not included in that review because no Japanese studies met the inclusion criteria. A study in Japan limited to 6,000 residents over 20 years old in a single city found that the prevalence of chronic pain was 61.6% among people aged 60–69, 72.1% for those in their 70 s, 56.4% for those in their 80 s, and 32.5% for those in their 90 s [2]. Another study among 13,217 residents over 40 years old in several municipalities was limited to anatomically localized pain. It found a prevalence of 20.9% for chronic lower back pain and 18.3% for chronic knee pain [3]. However, no large epidemiological studies of chronic pain have focused on community-dwelling older Japanese people.

Urbanization may also affect the prevalence and severity of chronic pain. A previous study in North Dakota reported that rural residents (i.e., those living in areas with a population of less than 2,500 people) showed a higher prevalence of chronic pain than urban residents [4]. Another study in North Carolina reported that rural residents with chronic low back pain had greater functional limitation and poorer function than urban residents with chronic low back pain [5]. However, the study included no definition of rural. For musculoskeletal concerns related to chronic pain, a systematic review concluded that hip fractures were more likely to be experienced by urban than rural residents [6]. However, no demographic studies of chronic pain have focused on Japanese residents.

The criteria used to define chronic widespread pain and chronic multisite pain vary, but the underlying clinical concepts are quite similar. They are included in the category of chronic primary pain in the 11th revision of the International Classification of Diseases (ICD-11) for 2022 [7–10]. A systematic review concluded that prevalence of chronic widespread pain in the general population was 10%–15%, and that it was higher in women and those over 40 years old [10]. Chronic widespread pain is a cardinal symptom of fibromyalgia, but it is also commonly observed in patients with several other diseases [7, 9, 11]. Previous research suggested that both chronic multisite pain and multisite pain among older people were associated with reductions in physical function [12, 13], psychological distress (e.g., anxiety and depressive symptoms) and socioeconomic factors [12, 13]. Chronic widespread pain and chronic multisite pain are important concepts in geriatric pain medicine, but there is little evidence about their prevalence among older Japanese people.

There is regional variation in healthy life expectancy in Japan [14]. However, no studies have explored regional variation in prevalence of chronic musculoskeletal pain. We hypothesized that regional variation would exist, and might be partly explained by urbanization and population aging because lifestyles vary significantly between metropolitan and rural areas.

This study used data from a large cohort study, the 2019 Japan Gerontological Evaluation Study (JAGES)[15], across 58 municipalities. We aimed to investigate the prevalence of chronic pain (overall chronic musculoskeletal pain, pain in each musculoskeletal part of the body, chronic widespread pain, and chronic multisite pain) in independent older Japanese people. We also investigated whether population density and population aging rate were associated with prevalence and municipal variation in chronic musculoskeletal pain using Bayesian multilevel regression analysis.

## Methods

### Study design and study population

This study was cross-sectional in design. In JAGES 2019, self-administered questionnaires were mailed to older residents (aged ≥ 65 years) identified from 2019 official residential registers of Japanese local governments. Residents who wished to participate completed and returned the questionnaire (response rate: 69.4%).

**Fig. 1:**
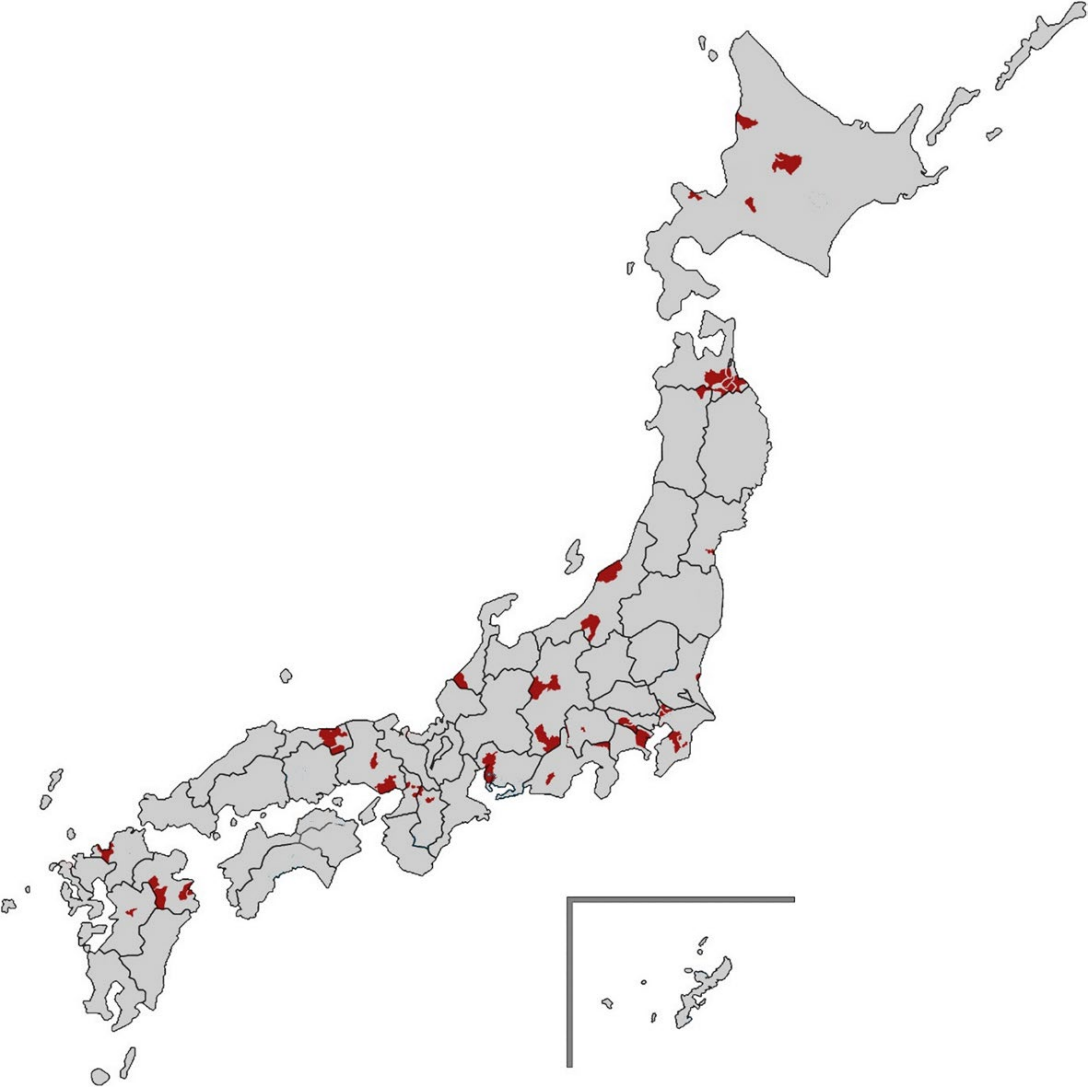
The 58 Japanese municipalities enrolled in this study. The study municipalities are shown in red on the map

In total, 24,342 participants who did not receive benefits from the national long-term care insurance completed the questionnaires, including items on their experience of chronic musculoskeletal pain. Supplementary Fig. 1 shows the enrollment process. We excluded 2,879 participants who needed support for daily living or were missing data on activities of daily living. We also excluded 10 participants missing data on municipality of residence, 6,049 participants missing data for pain items, 1,876 participants missing data for the Geriatric Depression Scale items, and 617 participants with history of cancer. We also excluded seven participants from one municipality because we considered that data from fewer than 10 residents would be insufficient for multilevel analysis. An additional 21 participants were excluded because they lived in a small village near Fukushima nuclear power plant, and we considered that the effects of evacuation might be potential confounders of the influence of population density and aging rate on chronic pain. This left a total of 12,883 community-dwelling older people (6,687 men and 6,196 women) living in 58 municipalities, and not in need of support for daily living. The 58 municipalities were dispersed across Japan, as shown in Fig. 1.

### Measures

#### Chronic musculoskeletal pain

We collected data about experience of pain in designated musculoskeletal sites (neck, shoulder, elbow, wrist, finger, back, lower back, hip, knee, ankle, and toe) lasting ≥ 3 months in the past year.

The 2019 JAGES questionnaire enabled us to identify lower back pain and knee pain separately from pain in other musculoskeletal parts of the body, using independent questions with a figure. The question for lower back pain was, “Did you have lower back pain (pain in the area shown in the figure) persisting a day or more in the past year? (yes or no)”. Those who answered yes were asked, “How long did you have lower back pain? (< 1 month, ≥ 1 month and < 3 months, or ≥ 3 months)”. We defined respondents with lower back pain lasting for ≥ 3 months in the past year as having chronic lower back pain. Similarly, the question for knee pain was, “Did you have knee pain (pain at the site shown in the figure) persisting a day or more in the past year? (yes or no)”. Those who answered yes were asked, “How long did you have knee pain? (< 1 month, ≥ 1 month and < 3 months, or ≥ 3 months)”. We defined respondents with knee pain lasting for ≥ 3 months in the past year as having chronic knee pain. For chronic pain in other musculoskeletal parts of the body, we used the single question, “Did you have any pain in other parts of the body lasting 3 months or more in the past year (multiple answers: none, neck, shoulder, elbow, wrist, finger, back, hip, ankle, and toe)?”.

The forthcoming ICD-11 defines chronic pain as persistent or recurrent pain lasting at least 3 months [8]. We therefore defined pain lasting ≥ 3 months in at least one of these areas as “chronic musculoskeletal pain”.

#### Chronic widespread-type pain and chronic multisite pain

There is no single agreed definition for chronic widespread pain [7]. We therefore created a new definition of “chronic widespread-type pain” as pain lasting ≥ 3 months in both the spinal area (i.e., neck, back, or lower back) and any peripheral area (i.e., shoulder, elbow, wrist, finger, hip, knee, ankle, and toe). This definition drew on two criteria for chronic widespread pain: the American College of Rheumatology (ACR) and the Manchester criteria. The ACR criteria suggest that widespread pain occurs in all body quadrants (on both sides of the body and above/below the waist; ‘side’ is considered to include right or left shoulder and buttock, so limb pain is not necessary), and also axially (cervical spine or anterior chest or thoracic spine or lumbar spine)[16]. The Manchester criteria define widespread pain as pain in at least two sections of two contralateral limbs (i.e., left arm/right leg or right arm/left leg) and axial skeletal pain (including the lower back) [17, 18].

The term “multisite pain”, or pain occurring simultaneously at multiple anatomical sites, is also used academically and clinically. Previous epidemiological studies found that classification of multiple anatomical sites could be based on the number of pain sites [19, 20]. We used the ACR suggestion that “pain in three sites (e.g., right shoulder, left buttock, and thoracic spine) qualifies as widespread pain” [16], and therefore created a new definition of “chronic multisite pain” as pain lasting ≥ 3 months in three or more anatomical sites.

#### Geriatric Depression Scale

To confirm the construct validity of our new definitions of chronic widespread-type pain and chronic multisite pain, we used the Geriatric Depression Scale (GDS). Previous studies compared widespread pain to depressive symptoms measured using the General Health Questionnaire [18, 21]. However, we selected the GDS because it was considered best for assessing depressive symptoms among older people in the general population. The Japanese version of GDS is used to assess the depressive symptoms of older people [22]. Its validity and reliability have previously been confirmed [22].

#### Demographic data

The questionnaire included questions about municipality of residence, age, gender, educational attainment (< 6 years, 6–9 years, 10–12 years, ≥ 13 years, and other), and marital status (married, widowed, divorced, single, and other).

Information about population size and municipality inhabitable area (ha) was taken from social and demographic statistics from 2018 [23], and national census data from 2019 [24]. Population density (people/km^2^) was obtained by dividing the overall population by municipal area (ha converted into km^2^). Population aging rate (%) was obtained by dividing the population aged 65 years and over by the overall population.

We used population density as a categorical variable for interpretability. We defined areas with a population density < 200 people/km^2^ as underpopulated, 200–1,999 people/km^2^ as suburban, 2000–3,999 people/km^2^ as urban, and ≥ 4000 people/km^2^ as metropolitan. These definitions were adapted from criteria used in analysis of designated zones for medical administration in Japan and the definition of densely inhabited districts (DID) used in the Japanese census [25, 26].

#### Health-related measures

The questionnaires asked about history of hypertension, diabetes mellitus, and hyperlipidemia (yes or no).

### Analytical procedure

Prevalence of each type of chronic pain was calculated by age group (65–69, 70–74, 75–79, 80–84, and ≥ 85 years old) and gender (men and women). We examined differences in depressive symptoms assessed using GDS by pain location, in line with previous studies using the General Health Questionnaire [15]. Age- and gender-adjusted means of GDS scores for each chronic pain distribution (peripheral area only, spinal area only, or chronic widespread-type pain [peripheral + spinal area]) and number of anatomical pain sites (1, 2, or ≥ 3, ranging from 1 to 11) were compared to no pain using analysis of covariance with Dunnett’s test. *P* for trend in GDS scores by number of anatomical pain sites was calculated using a general linear model. Municipal differences in age- and gender-adjusted prevalence for each type of chronic musculoskeletal pain were tested using analysis of covariance with Tukey’s test.

We investigated associations between age (5-year increments), gender, population density (< 200, 200–1,999, 2000–3,999, and ≥ 4000), and population aging rate (tertiles) and the prevalence and municipal variation in overall chronic musculoskeletal pain for all chronic pain types. Supplementary Fig. 2 shows the distribution of these factors among municipalities. Odds ratios (ORs) with 95% credible intervals (CI), median ORs, and the random parameters accounting for municipal variance in pain prevalence were estimated using Bayesian multilevel logistic regression analysis and the Markov Chain Monte Carlo (MCMC) method with 10,000 iterations. ORs were considered significant if the 95% CI did not include one. Model 1 included age (5-year increments) and gender. Model 2 added population density (< 200, 200–1,999, 2000–3,999, and ≥ 4000 person/km^2^). Model 3 added population aging rate (tertiles, %).

To determine the sample size for a multilevel logistic regression model, a previous simulation study concluded that an unbiased fixed effect parameters estimate was achieved by having ≥ 50 groups, each of at least 50 subjects, under maximum likelihood methods [27]. We had data from 58 (> 50) municipalities (i.e., groups). Of these 58 municipalities, 54 included > 50 residents (i.e., group size), and the remaining four municipalities included > 30 residents. As a sensitivity analysis, we also reran the Bayesian multilevel logistic regression analysis using the dataset that eliminated the four municipalities with fewer than 50 residents.

Bayesian multilevel logistic regression analysis used Stata Statistical Software: Release 16 (StataCorp LLC, 2019. College Station, TX, USA). Other statistical analyses used SAS, Version 9.4 (SAS Institute Inc., Cary, NC, USA).

## Results

Table 1 shows the characteristics of participants. Mean age was 73.6 (standard deviation 6.0) years old, and the study included approximately the same number of men (51.9%) and women (48.1%). The proportion of participants with < 10 years educational attainment was 19.6%, and 75.5% of participants were married. Population aging rate was ≥ 21.0% in all municipalities.

**Table 1:** Mean values and proportions for demographic factors (*n* = 12,883)

	**n**	**%**
**Number of municipalities**	58	-
**Age, years**		
**65–69**	3721	28.9
**70–74**	4004	31.1
**75–79**	2922	22.7
**80–84**	1565	12.1
**≥ 85**	671	5.2
**Gender**		
**Men**	6687	51.9
**Women**	6196	48.1
**Educational attainment**		
**Under 6 years**	40	0.3
**6–9 years**	2481	19.3
**10–12 years**	5696	44.2
**13 years and more**	4477	34.8
**Other**	78	0.6
**Missing**	111	0.9
**Marital status**		
**Married**	9731	75.5
**Widowed**	2007	15.6
**Divorced**	593	4.6
**Single**	413	3.2
**Other**	53	0.4
**Missing**	86	0.7
**History of hypertension**	5535	43.0
**History of diabetes**	1667	12.9
**History of hyperlipidemia**	2009	15.6
	**Mean**	**SD**
**GDS scores: 0–15**	2.8	2.9
**Population density, people/km**^**2**^** (municipality level)**	**n**	**%**
**< 200: underpopulated area**	650	5.0
**200–1,999: suburban area**	5,868	45.5
**2000–3,999: urban area**	970	7.5
**≥ 4000: metropolitan area**	5,395	41.9
**Population aging rate, % (municipality level)**		
**Minimum**	-	21.0
**Median**	-	25.7
**Maximum**	-	45.7
**First tertile: 21.0 to 24.3**	3,864	30.0
**Second tertile: 24.7 to 27.8**	4,939	38.3
**Third tertile: 28.4 to 45.7**	4,080	31.7

*GDS* Geriatric Depression Scale, *SD* Standard deviation

Table 2 shows the prevalence of each type of pain by age group and gender (ordering: chronic musculoskeletal pain, rank order of the prevalence of each pain site, chronic widespread-type pain, and chronic multisite pain). The prevalence of chronic musculoskeletal pain was 39.0% (men 36.3% and women 41.8%), and increased with age. The top three pain sites by prevalence were shoulder (14.6%), lower back (13.6%), and knee (11.8%). Supplementary Table 1 shows ranking of prevalence of pain site.

**Table 2:** Prevalence of chronic pain by age group and gender (*n* = 12,883)

Participant group	Age group (years)	Total		Men		Women	
		**n**	**%**	**n**	**%**	**n**	**%**
**All**	**65–69**	3721	100	1926	100	1795	100
	**70–74**	4004	100	2064	100	1940	100
	**75–79**	2922	100	1489	100	1433	100
	**80–84**	1565	100	842	100	723	100
	** ≥ 85**	671	100	366	100	305	100
	**Total**	12,883	100	6687	100	6196	100
**CMP**	**65–69**	1402	37.7	683	35.5	719	40.1
	**70–74**	1469	36.7	711	34.4	758	39.1
	**75–79**	1210	41.4	560	37.6	650	45.4
	**80–84**	655	41.9	334	39.7	321	44.4
	** ≥ 85**	284	42.3	140	38.3	144	47.2
	**Total**	5020	39.0	2428	36.3	2592	41.8
**Shoulder**	**65–69**	549	14.8	274	14.2	275	15.3
	**70–74**	582	14.5	290	14.1	292	15.1
	**75–79**	427	14.6	192	12.9	235	16.4
	**80–84**	240	15.3	120	14.3	120	16.6
	** ≥ 85**	88	13.1	40	10.9	48	15.7
	**Total**	1886	14.6	916	13.7	970	15.7
**Lower back**	**65–69**	432	11.6	233	12.1	199	11.1
	**70–74**	473	11.8	251	12.2	222	11.4
	**75–79**	465	15.9	227	15.2	238	16.6
	**80–84**	261	16.7	137	16.3	124	17.2
	** ≥ 85**	126	18.8	64	17.5	62	20.3
	**Total**	1757	13.6	912	13.6	845	13.6
**Knee**	**65–69**	367	9.9	149	7.7	218	12.1
	**70–74**	409	10.2	148	7.2	261	13.5
	**75–79**	380	13.0	134	9.0	246	17.2
	**80–84**	244	15.6	113	13.4	131	18.1
	** ≥ 85**	126	18.8	49	13.4	77	25.2
	**Total**	1526	11.8	593	8.9	933	15.1
**Neck**	**65–69**	237	6.4	136	7.1	101	5.6
	**70–74**	271	6.8	134	6.5	137	7.1
	**75–79**	205	7.0	109	7.3	96	6.7
	**80–84**	113	7.2	60	7.1	53	7.3
	** ≥ 85**	37	5.5	20	5.5	17	5.6
	**Total**	863	6.7	459	6.9	404	6.5
**Finger**	**65–69**	261	7.0	101	5.2	160	8.9
	**70–74**	260	6.5	99	4.8	161	8.3
	**75–79**	197	6.7	86	5.8	111	7.7
	**80–84**	97	6.2	39	4.6	58	8.0
	** ≥ 85**	32	4.8	11	3.0	21	6.9
	**Total**	847	6.6	336	5.0	511	8.2
**Hip**	**65–69**	213	5.7	95	4.9	118	6.6
	**70–74**	232	5.8	115	5.6	117	6.0
	**75–79**	206	7.0	91	6.1	115	8.0
	**80–84**	128	8.2	74	8.8	54	7.5
	** ≥ 85**	56	8.3	29	7.9	27	8.9
	**Total**	835	6.5	404	6.0	431	7.0
**Back**	**65–69**	158	4.2	73	3.8	85	4.7
	**70–74**	184	4.6	90	4.4	94	4.8
	**75–79**	156	5.3	66	4.4	90	6.3
	**80–84**	99	6.3	48	5.7	51	7.1
	** ≥ 85**	34	5.1	18	4.9	16	5.2
	**Total**	631	4.9	295	4.4	336	5.4
**Ankle**	**65–69**	117	3.1	44	2.3	73	4.1
	**70–74**	120	3.0	50	2.4	70	3.6
	**75–79**	125	4.3	48	3.2	77	5.4
	**80–84**	86	5.5	36	4.3	50	6.9
	** ≥ 85**	32	4.8	18	4.9	14	4.6
	**Total**	480	3.7	196	2.9	284	4.6
**Wrist**	**65–69**	138	3.7	53	2.8	85	4.7
	**70–74**	118	2.9	47	2.3	71	3.7
	**75–79**	92	3.1	35	2.4	57	4.0
	**80–84**	52	3.3	26	3.1	26	3.6
	** ≥ 85**	34	5.1	11	3.0	23	7.5
	**Total**	434	3.4	172	2.6	262	4.2
**Toe**	**65–69**	99	2.7	39	2.0	60	3.3
	**70–74**	105	2.6	42	2.0	63	3.2
	**75–79**	102	3.5	38	2.6	64	4.5
	**80–84**	75	4.8	36	4.3	39	5.4
	** ≥ 85**	23	3.4	13	3.6	10	3.3
	**Total**	404	3.1	168	2.5	236	3.8
**Elbow**	**65–69**	112	3.0	69	3.6	43	2.4
	**70–74**	98	2.4	59	2.9	39	2.0
	**75–79**	91	3.1	53	3.6	38	2.7
	**80–84**	52	3.3	35	4.2	17	2.4
	** ≥ 85**	24	3.6	11	3.0	13	4.3
	**Total**	377	2.9	227	3.4	150	2.4
**CWTP**	**65–69**	455	12.2	218	11.3	237	13.2
	**70–74**	492	12.3	231	11.2	261	13.5
	**75–79**	469	16.1	197	13.2	272	19.0
	**80–84**	262	16.7	129	15.3	133	18.4
	** ≥ 85**	119	17.7	55	15.0	64	21.0
	**Total**	1797	13.9	830	12.4	967	15.6
**CMSP**	**65–69**	338	9.1	154	8.0	184	10.3
	**70–74**	360	9.0	160	7.8	200	10.3
	**75–79**	328	11.2	131	8.8	197	13.7
	**80–84**	206	13.2	98	11.6	108	14.9
	** ≥ 85**	89	13.3	38	10.4	51	16.7
	**Total**	1321	10.3	581	8.7	740	11.9

*CMP* Chronic musculoskeletal pain, *CWTP* Chronic widespread-type pain, *CMSP* Chronic multisite pain

Chronic widespread-type pain was defined as chronic pain in both the spinal area (i.e., neck, back, or lower back) and any peripheral area

Chronic multisite pain was defined as chronic pain in three or more sites

Proportions were adjusted for age and gender

The prevalence of chronic musculoskeletal pain by category of population density was 39.1% for underpopulated, 39.6% for suburban, 35.9% for urban, and 38.8% for metropolitan areas. For chronic multisite-type pain, the corresponding figures were 14.8%, 14.3%, 13.7%, and 13.6%, and for chronic multisite pain we found 11.6%, 10.3%, 9.1%, and 10.3% (not shown in the table).

Table 3 shows adjusted mean GDS scores by pain distribution and number of pain sites. Participants with pain in only peripheral areas or spinal areas had higher GDS scores than those without pain. Participants with chronic widespread-type pain had the highest GDS scores. GDS score increased with number of pain sites (0, 1, 2, ≥ 3) in a dose–response manner (*p* for trend < 0.001). Supplementary Table 2 shows the full results for anatomical pain sites.

**Table 3:** Pain location and Geriatric Depression Scale (GDS) scores (*n* = 12,883)

	**Number**	**(%)**	**GDS score**		
			**Adjusted mean**	**SE**	***P* value**
**Pain distribution**					
**No pain**	7863	61.0	2.4	0.03	(reference)
**Peripheral area only**	2483	19.3	3.2	0.06	< .0001
**Spinal area only**	740	5.7	3.3	0.10	< .0001
**CWTP (Peripheral + spinal area)**	1797	13.9	4.0	0.07	< 0.001
**Number of pain sites**					
**0**	7863	61	2.4	0.03	(reference)
**1**	2390	18.6	3.1	0.06	< 0.001
**2**	1309	10.2	3.5	0.08	< 0.001
**≥ 3 (CMSP)**	1321	10.3	4.3	0.08	< 0.001
			*P* for trend < 0.001		

*GDS* Geriatric depression scale, *SE* Standard error, *CWTP* Chronic widespread-type pain, CMSP: chronic multisite pain. Means of GDS scores were adjusted for age and gender. *P* value was tested using analysis of covariance. *P* for linear trend was calculated using a general linear model

*P* for linear trend < 0.001

**Fig. 2:**
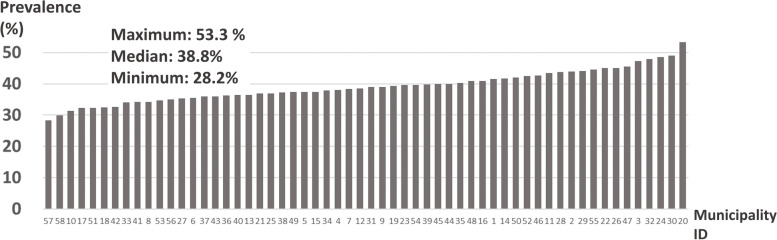
Prevalence of overall chronic musculoskeletal pain by municipality. The X-axis shows municipality IDs, and the Y-axis shows the prevalence of chronic musculoskeletal pain (%). The maximum, median, and minimum prevalence were 53.3%, 38.8%, 28.2%

Chronic musculoskeletal pain prevalence by municipality is shown in Fig. 2. This varied significantly across municipalities (*p* < 0.001), ranging from 28.2% to 53.3%, with a median of 38.8%. Supplementary Table 3 and Supplementary Fig. 3 show the prevalence of pain in specific anatomical pain sites, by population, inhabitable area, and population density of municipality. Significant differences were found between municipalities for pain in neck (*p* = 0.01), shoulder (*p* = 0.02), knee (*p* = 0.02), and toe (*p* = 0.01).

Table 4 shows ORs (95% CI) and median ORs of prevalence of chronic musculoskeletal pain with between-municipality variance. There was a higher prevalence in older people and women. Aging and gender contributed 3.4% to between-municipality variance. People living in underpopulated, suburban, and metropolitan areas were more likely to have chronic musculoskeletal pain than those in urban areas, although this was not statistically significant. Median ORs for underpopulated, suburban, and metropolitan areas were 1.15, 1.16, and 1.16. However, population density did not explain between-municipality variance in chronic musculoskeletal pain. Population aging rate did not explain either prevalence or between-municipality variance in chronic musculoskeletal pain. The results of the sensitivity analysis for the ORs (95% CI) and median ORs of prevalence of chronic musculoskeletal pain with between-municipality variance, using the dataset eliminating the four municipalities with fewer than 50 residents, were similar to these results.

**Table 4:** Odds ratios (95% credible intervals) and median odds ratios of prevalence of chronic pain: the results of Bayesian multilevel logistic regression analysis

	**Null model:**	**Model 1: Individual variables**			**Model 2: Added population density**			**Model 3: Added population aging rate**		
		**OR**	**95% CI**	**MOR**	**OR**	**95% CI**	**MOR**	**OR**	**95% CI**	**MOR**
**Age, 5 year increments**		**1.07**	**1.04–1.11**	**1.07**	**1.07**	**1.04–1.11**	**1.08**	**1.07**	**1.04–1.11**	**1.07**
**Women (ref. men)**		**1.27**	**1.18–1.36**	**1.27**	**1.27**	**1.18–1.35**	**1.27**	**1.27**	**1.18–1.36**	**1.27**
**Population density, people/km**^**2**^										
** < 200: underpopulated area**					1.15	0.86–1.51	1.13	1.06	0.79–1.35	1.05
**200–1,999: suburban area**					1.17	0.93–1.43	1.16	1.06	0.91–1.25	1.05
**2000–3,999: urban area**					1 (reference)			1 (reference)		
**≥ 4000: metropolitan area**					1.17	0.94–1.46	1.16	1.07	0.91–1.27	1.16
**Population aging rate**										
**First tertile**								1 (reference)		
**Second tertile**								1.02	0.89–1.14	1.02
**Third tertile**								1.04	0.87–1.21	1.03
**Between-municipality variance**	0.0176 (0.0004)	0.0170 (0.0005)			0.0187 (0.0005)			0.0189 (0.0007)		
**Proportional changes variance**		+ 3.4% (vs. Null model)			− 10.0% (vs. Model 1)			− 1.1% (vs. Model 2)		
**Median odds ratio between municipalities**	1.13	1.13			1.14			1.14		

*CI* Credible interval, *CMP* Chronic musculoskeletal pain, *MCSE* Monte Carlo standard error, *MOR* Median odds ratio, *OR* Odds ratio. Bayesian multilevel logistic regression model was used to estimate the random parameters accounting for variation between municipalities. Statistically significant results are shown in bold

Model 1: Adjusted for age and gender

Model 2: Adjusted for age, gender, and population density

Model 3: Adjusted for age, gender, population density, and population aging rate

## Discussion

The population aging rate in all 58 municipalities studied was ≥ 21.0%, the World Health Organization and United Nations definition of a super-aged society [28]. We found a prevalence of 39.0% (men 36.3%, women 41.8%) for chronic musculoskeletal pain, 13.9% (men 12.4%, women 15.6%) for chronic widespread-type pain, and 10.3% (men 8.7%, women 11.9%) for chronic multisite pain. The top three anatomical sites by prevalence were shoulder, lower back, and knee. Individuals with chronic widespread-type or chronic multisite pain were more likely than individuals without pain to show high levels of depressive symptoms. Older people and women had a higher prevalence of chronic musculoskeletal pain. People living in underpopulated, suburban, or metropolitan areas tended to have more chronic musculoskeletal pain than those in urban areas, but this was not statistically significant. Population density and population aging rate were not associated with differences between municipalities.

A previous study in a single Japanese city found chronic pain prevalence of over 50% among those in their 60 s–80 s [2], which was higher than our figure of 39% for chronic musculoskeletal pain. This may be because that study included chronic pain other than musculoskeletal pain, such as headaches and abdominal pain.

Residential environmental factors related to population density that may affect chronic musculoskeletal pain prevalence include city design, geographical situation, and climate. For example, many Japanese provinces are hilly. A previous study found neighborhood walkability was associated with knee pain in older people [29]. There may also be differences in medical care between urban and underpopulated areas that may affect chronic musculoskeletal pain prevalence.

To our knowledge, this is the first study to report on prevalence of chronic widespread-type or chronic multisite pain in independent older Japanese people. There is no consistent definition of chronic widespread pain or chronic multisite pain. We therefore defined *chronic widespread-type pain* as pain experienced in both the spinal area and at least one peripheral area, and *chronic multisite pain* as pain experienced in ≥ three anatomical sites, drawing on several previous criteria. Individuals meeting our definition of chronic widespread pain and chronic multisite pain were more likely to have depressive symptoms than those without pain. This result is consistent with previous studies [18, 21], suggesting that our definitions were valid.

Population density was not associated with municipal variation in chronic musculoskeletal pain prevalence. Further research is needed to explore additional factors that contribute to municipal variation in chronic musculoskeletal pain prevalence, such as socioeconomic factors [30].

Chronic pain has negative effects on ability to perform activities of daily living and may increase the burden on caregivers [31]. As the number of older people with chronic pain increases, so will the social burden. Policy-makers and healthcare providers should consider developing a strategy on chronic pain as we move toward a super-aging society.

The strength of our study was using data from a large cohort study coordinated with local governments. However, this study also had several limitations. First, we examined only 58 of 1,718 municipalities in Japan, and the results may therefore not be fully representative. However, the mean rate of population aging in our study (27.8%) was similar to the rate of 28.4% reported for Japan as a whole in 2019 [24]. Second, the dataset for JAGES excluded residents who received national long-term care insurance benefits. We therefore excluded participants who needed support for daily living even if they did not receive benefits. The major reasons for needing long-term care insurance benefits among older Japanese residents are *dementia* and *musculoskeletal disorders* [32]. It is difficult for older people with dementia to respond accurately to self-reported questionnaires. We therefore excluded anyone receiving long-term care benefits or needing support for daily living. However, this population has a higher level of comorbidities, including musculoskeletal disorders related to disability and pain. Older people with disabilities that resulted from severe chronic musculoskeletal pain could therefore have been excluded from the analyses, so our results may underestimate chronic musculoskeletal pain prevalence in community-dwelling people. An external framework should be established in the future to investigate older people who received benefits from the national long-term care insurance or were in long-care facilities and hospitals. Third, we did not have information about treatment or medication for pain. Prescribed or over-the-counter analgesics may suppress pain symptoms, which could have influenced the results. Finally, we did not collect data about chronic pain other than musculoskeletal pain. Headache, orofacial pain, and visceral pain are also important health problems among older people. However, chronic musculoskeletal pain is the dominant type of chronic pain for all ages, and directly influences physical mobility. We therefore believe it is important to understand chronic musculoskeletal pain prevalence among independent older people to inform health policy.

## Conclusions

Chronic musculoskeletal pain prevalence in independent older Japanese people in this study was consistent with previous global reports. Population density tended to be associated with chronic musculoskeletal pain prevalence, although this was not statistically significant, and did not explain municipal variation. Population aging rate was also not associated with chronic musculoskeletal pain prevalence and municipal variation. Further research is needed to investigate other factors that contribute to regional variance. Our findings may inform healthcare policy for chronic pain in older people, and add to the evidence about environmental factors affecting chronic pain.

## Supplementary Information

**Additional file 1: Figure S1.** Enrollment process for participants. **Table S1.** Rankings of pain sites. **Table S2.** Number of pain sites and Geriatric Depression Scale (GDS) scores (*n* = 12,883). **Table S3.** Demographic factors and prevalence of chronic pain among participants by municipality (*n* = 58). **Figure S2.** Distribution of age, gender, population density, and aging rate. The Xaxis indicates municipality IDs. The Y-axes indicate means of age (year), proportion of women (%), population density (people/km2 ), and aging rate (%). **Figure S3.** Prevalence of each type of chronic pain. The X-axis indicates the prevalence of each type of chronic musculoskeletal pain (CMP) (%), and the Y-axis indicates municipality IDs. The maximum, median, and minimum prevalence of CMP are also indicated.

## Data Availability

All enquiries should be addressed to the data management committee via e-mail: dataadmin.ml@jages.net. All JAGES datasets have ethical or legal restrictions for public deposition owing to inclusion of sensitive information from the participants. Following the regulation of the local governments that cooperated with our survey, the JAGES data management committee has imposed restrictions upon the data.

## Abbreviations

ICD-11: 11th revision of the International Classification of Diseases
JAGES: Japan Gerontological Evaluation Study
ACR: American College of Rheumatology
GDS: Geriatric Depression Scale
OR: Odds ratio
CI: Credible intervals
MCMC: Markov Chain Monte Carlo
