# Supplementary material for: Prevalence and municipal variation in chronic musculoskeletal pain among independent older people: data from the Japan Gerontological Evaluation Study (JAGES)
Source: BMC Musculoskelet Disord. 2022 Aug 5;23:755. doi: 10.1186/s12891-022-05694-y (PMC9356514; doi:10.1186/s12891-022-05694-y)
Supplement: Supplementary file 1 — Additional file 1: Figure S1. Enrollment process for participants. Table S1. Rankings of pain sites. Table S2. Number of pain sites and Geriatric Depression Scale (GDS) scores (n = 12,883). Table S3. Demographic factors and prevalence of chronic pain among participants by municipality (n = 58). Figure S2. Distribution of age, gender, population density, and aging rate. The Xaxis indicates municipality IDs. The Y-axes indicate means of age (year), proportion of women (%), population density (people/km2 ), and aging rate (%). Figure S3. Prevalence of each type of chronic pain. The X-axis indicates the prevalence of each type of chronic musculoskeletal pain (CMP) (%), and the Y-axis indicates municipality IDs. The maximum, median, and minimum prevalence of CMP are also indicated. [file 12891_2022_5694_MOESM1_ESM.pdf]

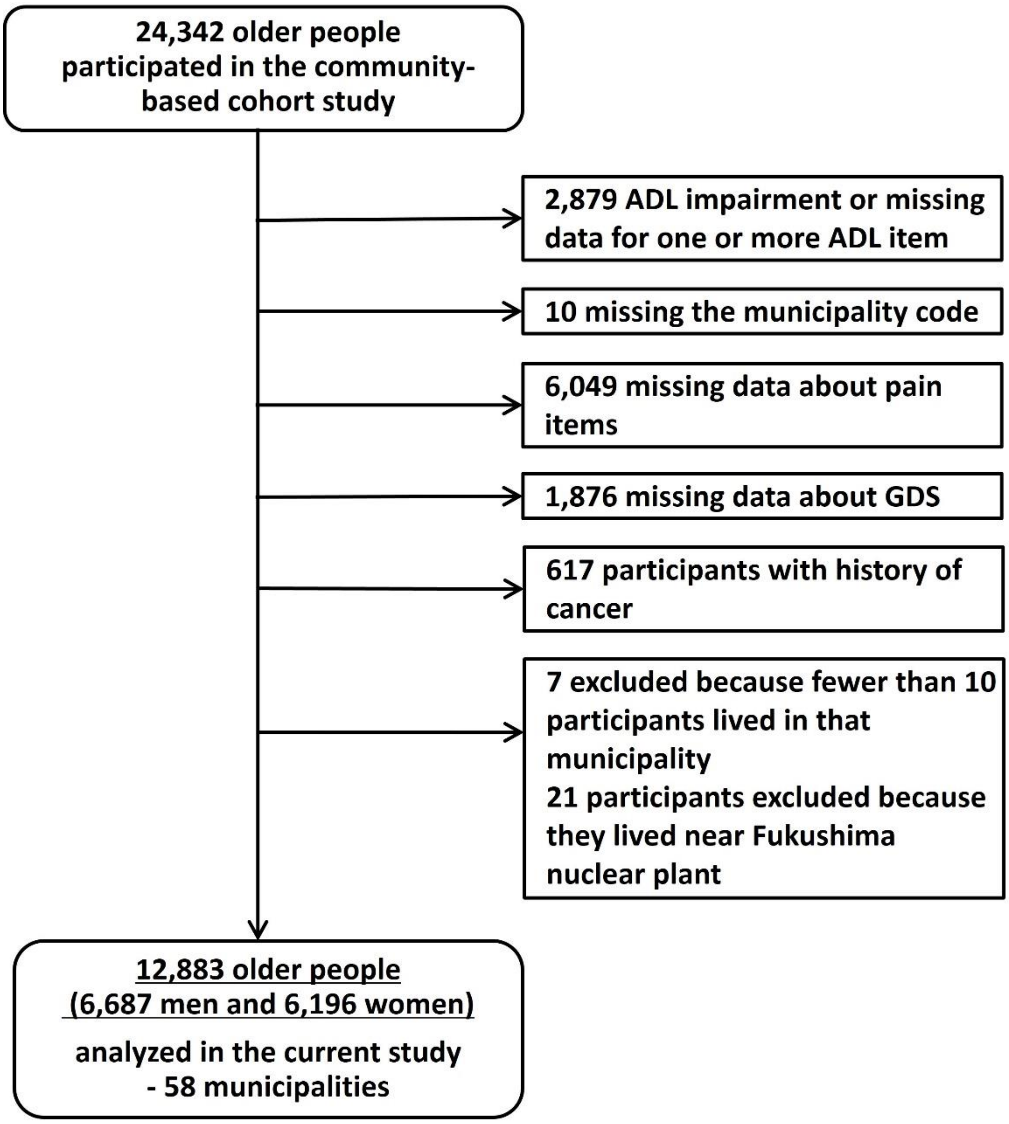

Supplementary Figure 1. Enrollment process for participants.

Supplementary Table 1. Rankings of pain sites

Rank	All participants (%)		Men (%)		Women (%)	
**#1**	Shoulder	14.6	Shoulder	13.7	Shoulder	15.7
**#2**	Lower back	13.6	Lower back	13.6	Knee	15.1
**#3**	Knee	11.8	Knee	8.9	Lower back	13.6
**#4**	Neck	6.7	Neck	6.9	Finger	8.2
**#5**	Finger	6.6	Hip	6.0	Hip	7.0
**#6**	Hip	6.5	Finger	5.0	Neck	6.5
**#7**	Back	4.9	Back	4.4	Back	5.4
**#8**	Ankle	3.7	Elbow	3.4	Ankle	4.6
**#9**	Wrist	3.4	Ankle	2.9	Wrist	4.2
**#10**	Toe	3.1	Wrist	2.6	Toe	3.8
**#11**	Elbow	2.9	Toe	2.5	Elbow	2.4

Proportions were adjusted for age and gender.

Supplementary Table 2. Number of pain sites and Geriatric Depression Scale (GDS) scores (n = 12,883)

			GDS score		
	Number	(%)	Adjusted mean	SE	P value
**Number of pain sites**					
**0**	7863	61.0	2.4	0.032	(reference)
**1**	2390	18.6	3.1	0.057	<0.001
**2**	1309	10.2	3.5	0.078	<0.001
**3**	726	5.6	4.1	0.10	<0.001
**4**	331	2.6	4.2	0.154	<0.001
**5**	140	1.1	4.9	0.237	<0.001
**6**	77	0.6	5.3	0.32	<0.001
**7**	24	0.2	4.3	0.572	<0.001
**8**	13	0.1	6.3	0.778	<0.001
**9**	6	0.05	4.0	1.145	0.86
**10**	2	0.02	9.0	1.982	0.01
**11**	2	0.02	3.5	1.983	1.00
			**P for trend <0.001**		

Abbreviations: GDS: geriatric depression scale, SE: standard error.

Supplementary Table 3. Demographic factors and prevalence of chronic pain among participants by municipality (n = 58)

Municipal ID	Number of participants	Population	Inhabitable area (ha)	Population density (person/km^2)	Population aging rate	CMP	CWTP	CMSP
1	86	7337	2586	284	38.1%	41.5%	15.0%	13.8%
2	159	23309	6814	342	35.4%	43.9%	17.1%	12.6%
3	46	10135	5500	184	39.6%	47.2%	12.5%	8.2%
4	107	67398	5238	1287	25.7%	38.0%	18.4%	14.5%
5	1172	2300000	31505	7287	24.7%	37.3%	11.8%	9.5%
6	77	10233	5394	190	26.0%	35.5%	12.2%	9.5%
7	74	8111	7448	109	32.2%	38.3%	15.3%	11.2%
8	111	10292	31419	33	37.5%	34.2%	12.6%	9.0%
9	175	19607	3953	496	38.7%	39.0%	15.2%	9.5%
10	32	3265	9033	36	41.6%	31.2%	12.5%	15.6%
11	107	12344	9321	132	39.2%	43.4%	17.4%	9.9%
12	212	63429	20506	309	32.2%	38.5%	12.7%	12.7%
13	66	5339	1905	280	43.3%	36.4%	13.7%	12.1%
14	72	19497	4408	442	29.1%	41.7%	12.6%	12.5%
15	332	71346	3668	1945	23.2%	37.4%	16.0%	10.6%
16	202	275000	23326	1178	28.4%	40.9%	14.2%	9.2%
17	98	67186	9182	732	33.8%	32.2%	9.7%	5.5%
18	77	143000	1271	11254	28.8%	32.4%	10.5%	9.1%
19	53	124000	1230	10047	29.3%	39.3%	9.4%	15.0%
20	36	56075	1125	4984	26.7%	53.3%	31.0%	19.8%

21	170	17969	2995	600	42.6%	36.8%	12.3%	9.3%
22	58	9645	4585	210	41.8%	45.0%	19.2%	10.5%
23	115	22332	13561	165	45.7%	39.5%	18.5%	17.6%
24	321	243000	24307	1001	27.6%	48.5%	15.7%	10.2%
25	247	31124	2617	1189	24.2%	36.9%	14.6%	8.9%
26	70	7154	1671	428	39.8%	45.0%	16.3%	13.4%
27	37	16886	1827	924	32.0%	35.2%	16.4%	13.6%
28	169	17237	4326	399	34.0%	43.7%	19.5%	15.4%
29	170	395000	3637	10874	25.6%	44.0%	14.1%	11.1%
30	273	102000	12619	805	31.6%	49.0%	16.1%	12.0%
31	453	44678	4647	961	25.5%	39.0%	12.3%	9.4%
32	59	17433	8489	205	37.8%	47.9%	17.8%	12.8%
33	290	42473	2320	1831	24.8%	34.1%	13.2%	9.7%
34	386	117000	4606	2538	23.8%	37.8%	14.9%	10.7%
35	346	56547	5120	1104	25.5%	40.2%	11.2%	9.5%
36	240	112000	4259	2628	21.9%	36.3%	14.1%	6.2%
37	215	89157	3257	2737	21.2%	35.9%	13.5%	9.8%
38	264	84617	4338	1951	27.1%	37.1%	14.4%	8.0%
39	131	49230	2950	1669	25.3%	39.8%	14.5%	7.7%
40	216	194000	21271	911	28.4%	36.4%	12.8%	8.6%
41	91	21200	3557	596	35.1%	34.1%	9.8%	5.2%
42	86	18528	3874	478	33.0%	32.7%	10.9%	8.6%
43	50	18312	7563	242	37.0%	35.9%	14.1%	8.0%
44	120	38748	8393	462	39.0%	39.9%	13.3%	9.9%
45	371	482000	5604	8596	21.0%	39.9%	11.4%	7.9%

46	234	432000	6387	6769	26.7%	42.7%	14.9%	9.8%
47	548	1540000	33158	4636	27.8%	45.5%	15.6%	12.5%
48	185	231000	20331	1138	29.5%	40.9%	16.5%	7.8%
49	259	1260000	21278	5940	22.9%	37.3%	16.4%	12.9%
50	88	10423	5655	184	32.3%	41.9%	13.6%	11.3%
51	271	810000	67048	1208	28.7%	32.3%	12.4%	11.3%
52	244	414000	10133	4085	25.7%	42.4%	11.7%	7.6%
53	417	578000	10651	5422	26.5%	34.6%	11.1%	8.4%
54	837	1540000	23174	6640	21.5%	39.7%	16.2%	11.6%
55	371	54917	18947	290	37.9%	44.6%	16.1%	12.1%
56	977	3720000	39941	9326	24.3%	35.0%	13.3%	10.0%
57	81	7222	2221	325	39.2%	28.2%	9.9%	9.8%
58	129	118000	3369	3509	27.4%	29.9%	9.7%	8.1%
						**CMP**	**WTP**	**MSP**
					**P for difference**	< 0.001	0.40	0.41

**Municipal ID**	**Neck**	**Shoulder**	**Elbow**	**Wrist**	**Finger**	**Back**	**Lower back**	**Hip**	**Knee**	**Ankle**	**Toe**
1	4.7%	16.1%	4.7%	2.2%	4.4%	4.6%	19.9%	5.8%	13.6%	-0.1%	3.4%
2	6.3%	18.7%	3.2%	4.3%	7.9%	5.1%	14.8%	10.8%	10.1%	3.1%	3.1%
3	2.2%	19.5%	4.4%	6.4%	10.8%	10.7%	12.5%	4.1%	14.4%	-0.2%	6.4%
4	8.4%	14.9%	4.7%	7.5%	7.4%	4.9%	15.7%	8.8%	13.9%	4.0%	3.9%
5	5.9%	13.8%	2.4%	2.9%	6.6%	5.3%	11.4%	6.0%	10.6%	4.6%	3.1%
6	9.1%	16.8%	2.7%	2.6%	10.2%	4.0%	13.6%	2.9%	9.7%	0.2%	1.4%
7	2.7%	13.5%	2.7%	2.7%	9.4%	2.8%	15.4%	11.1%	11.5%	4.2%	1.5%

8	5.4%	11.7%	0.9%	3.6%	7.2%	4.5%	10.9%	5.4%	10.8%	8.1%	3.6%
9	4.6%	14.7%	2.4%	2.2%	6.6%	4.5%	15.0%	9.1%	11.6%	4.5%	2.2%
10	3.1%	9.4%	3.1%	6.3%	6.3%	0.0%	15.5%	12.5%	18.7%	3.1%	0.0%
11	5.6%	14.9%	2.8%	1.8%	6.4%	4.5%	17.5%	7.3%	14.3%	4.5%	3.6%
12	3.8%	14.1%	3.8%	6.1%	9.3%	2.8%	12.4%	6.1%	11.6%	4.7%	3.3%
13	6.1%	19.7%	3.0%	0.0%	3.1%	4.6%	15.1%	6.1%	15.2%	7.6%	3.0%
14	11.1%	11.1%	2.8%	1.4%	6.9%	4.2%	19.6%	5.6%	7.0%	2.8%	2.8%
15	9.0%	14.8%	3.3%	4.8%	5.4%	6.6%	14.5%	5.7%	11.5%	3.3%	1.8%
16	7.9%	11.9%	4.4%	3.5%	4.0%	5.0%	16.6%	6.1%	12.3%	5.6%	3.6%
17	0.0%	9.2%	1.0%	4.2%	8.2%	4.2%	11.7%	4.3%	11.0%	5.3%	1.2%
18	3.9%	6.4%	4.0%	3.8%	5.0%	3.9%	13.3%	9.2%	7.8%	2.6%	7.8%
19	7.6%	18.6%	2.0%	1.7%	7.2%	1.9%	6.0%	11.4%	11.1%	3.7%	9.4%
20	13.9%	25.1%	2.7%	2.9%	8.5%	8.5%	22.5%	5.7%	22.9%	3.0%	8.5%
21	6.5%	12.2%	2.4%	4.0%	2.7%	4.1%	11.9%	11.2%	9.8%	3.5%	3.5%
22	5.2%	22.3%	5.2%	5.2%	5.1%	5.2%	26.2%	10.5%	8.9%	1.8%	1.8%
23	3.5%	16.6%	6.0%	6.2%	8.0%	3.6%	18.3%	13.1%	11.8%	2.7%	3.6%
24	7.8%	16.9%	3.1%	1.9%	9.1%	6.5%	14.8%	7.7%	12.9%	3.1%	5.0%
25	4.4%	12.2%	4.0%	2.9%	4.5%	4.6%	15.7%	5.1%	13.2%	3.8%	3.4%
26	5.7%	20.2%	4.2%	3.0%	1.7%	7.3%	13.1%	8.8%	19.5%	3.1%	0.2%
27	5.4%	10.7%	0.1%	5.4%	7.9%	5.5%	11.2%	8.2%	24.5%	2.8%	8.1%
28	11.9%	23.5%	4.2%	4.7%	5.1%	5.9%	16.8%	7.2%	15.3%	6.5%	0.6%
29	9.4%	20.6%	3.5%	3.5%	8.8%	4.7%	10.0%	5.9%	12.2%	2.3%	3.5%
30	11.0%	16.4%	3.3%	4.8%	8.0%	6.3%	18.3%	7.1%	14.7%	4.5%	1.9%
31	3.8%	13.2%	2.2%	3.1%	5.5%	4.0%	12.7%	6.2%	15.0%	5.1%	4.2%
32	6.8%	15.0%	6.9%	6.5%	13.2%	3.1%	18.1%	-0.4%	12.1%	6.4%	1.4%

33	6.2%	15.0%	2.5%	2.3%	6.0%	3.5%	13.1%	6.3%	11.4%	1.4%	1.4%
34	8.5%	13.2%	3.6%	4.5%	8.1%	5.3%	14.4%	3.8%	9.4%	3.8%	2.7%
35	4.6%	16.5%	3.4%	4.7%	6.8%	5.9%	13.7%	6.7%	9.9%	2.7%	3.3%
36	6.7%	13.9%	3.7%	3.0%	6.8%	2.5%	11.8%	4.5%	10.4%	2.5%	2.1%
37	6.0%	13.1%	5.1%	4.2%	3.9%	4.7%	12.4%	5.5%	10.3%	1.9%	3.3%
38	4.5%	13.6%	2.7%	2.3%	4.9%	4.6%	13.7%	6.8%	12.9%	2.3%	3.4%
39	6.9%	18.4%	0.0%	2.3%	7.0%	3.1%	12.9%	5.3%	8.5%	3.8%	4.6%
40	7.0%	14.8%	2.8%	2.3%	3.1%	6.1%	12.4%	8.0%	10.6%	2.4%	1.0%
41	3.3%	11.1%	1.1%	1.3%	3.5%	5.8%	9.6%	8.2%	10.3%	3.7%	1.4%
42	7.0%	12.5%	3.6%	5.5%	3.0%	3.3%	12.6%	3.2%	5.7%	2.0%	2.1%
43	8.0%	15.8%	2.1%	1.9%	9.7%	2.0%	16.5%	0.2%	8.0%	6.0%	0.0%
44	4.2%	14.1%	1.7%	3.3%	6.6%	4.1%	14.2%	9.1%	15.7%	4.1%	3.3%
45	7.3%	13.1%	1.4%	3.2%	7.6%	4.1%	11.0%	5.2%	14.9%	5.1%	3.2%
46	10.3%	21.0%	1.3%	3.4%	6.4%	2.5%	14.0%	6.4%	9.7%	3.0%	1.7%
47	7.8%	17.4%	3.6%	3.1%	7.6%	4.7%	15.1%	6.2%	13.8%	5.9%	6.5%
48	9.7%	16.3%	3.8%	1.7%	8.2%	5.5%	14.3%	7.2%	9.6%	3.4%	1.2%
49	8.5%	15.1%	1.9%	2.4%	7.4%	5.5%	16.4%	8.2%	14.6%	2.0%	3.9%
50	4.6%	12.4%	5.7%	4.5%	6.6%	5.7%	17.3%	5.7%	16.9%	4.5%	1.1%
51	7.0%	12.6%	2.2%	2.6%	3.7%	2.9%	11.6%	7.3%	7.9%	5.1%	2.2%
52	4.9%	17.2%	2.0%	5.7%	9.5%	4.8%	11.6%	5.2%	9.1%	4.8%	4.4%
53	6.5%	12.5%	2.9%	2.6%	5.0%	3.9%	12.0%	4.8%	9.0%	2.7%	3.9%
54	7.5%	15.2%	2.9%	3.2%	6.1%	6.6%	15.3%	7.5%	13.7%	3.3%	2.8%
55	7.8%	16.8%	2.9%	2.5%	8.0%	5.7%	15.4%	6.0%	16.3%	3.3%	3.3%
56	6.8%	11.5%	2.4%	3.3%	6.9%	5.6%	12.3%	5.9%	10.2%	3.4%	2.9%
57	2.5%	8.5%	6.3%	1.1%	8.4%	4.9%	7.7%	7.5%	7.3%	2.4%	4.9%

58	6.2%	10.1%	3.1%	4.7%	6.2%	2.5%	7.4%	4.9%	10.7%	1.7%	1.7%
	**Neck**	**Shoulder**	**Elbow**	**Wrist**	**Finger**	**Back**	**Lower back**	**Hip**	**Knee**	**Ankle**	**Toe**
**P for difference**	0.01	0.02	0.74	0.59	0.35	0.91	0.21	0.20	0.02	0.22	0.01

Abbreviations: CMP: chronic musculoskeletal pain, CWTP: chronic widespread-type pain, CMSP: chronic multisite pain.

Chronic widespread-type pain was defined as chronic pain in both the spinal area (i.e., neck, back, or lower back) and any peripheral area.

Chronic multisite pain was defined as chronic pain in three or more sites.

Proportions other than population aging rate were adjusted for age and gender.

P for difference was calculated using analysis of covariance with Tukey's test.

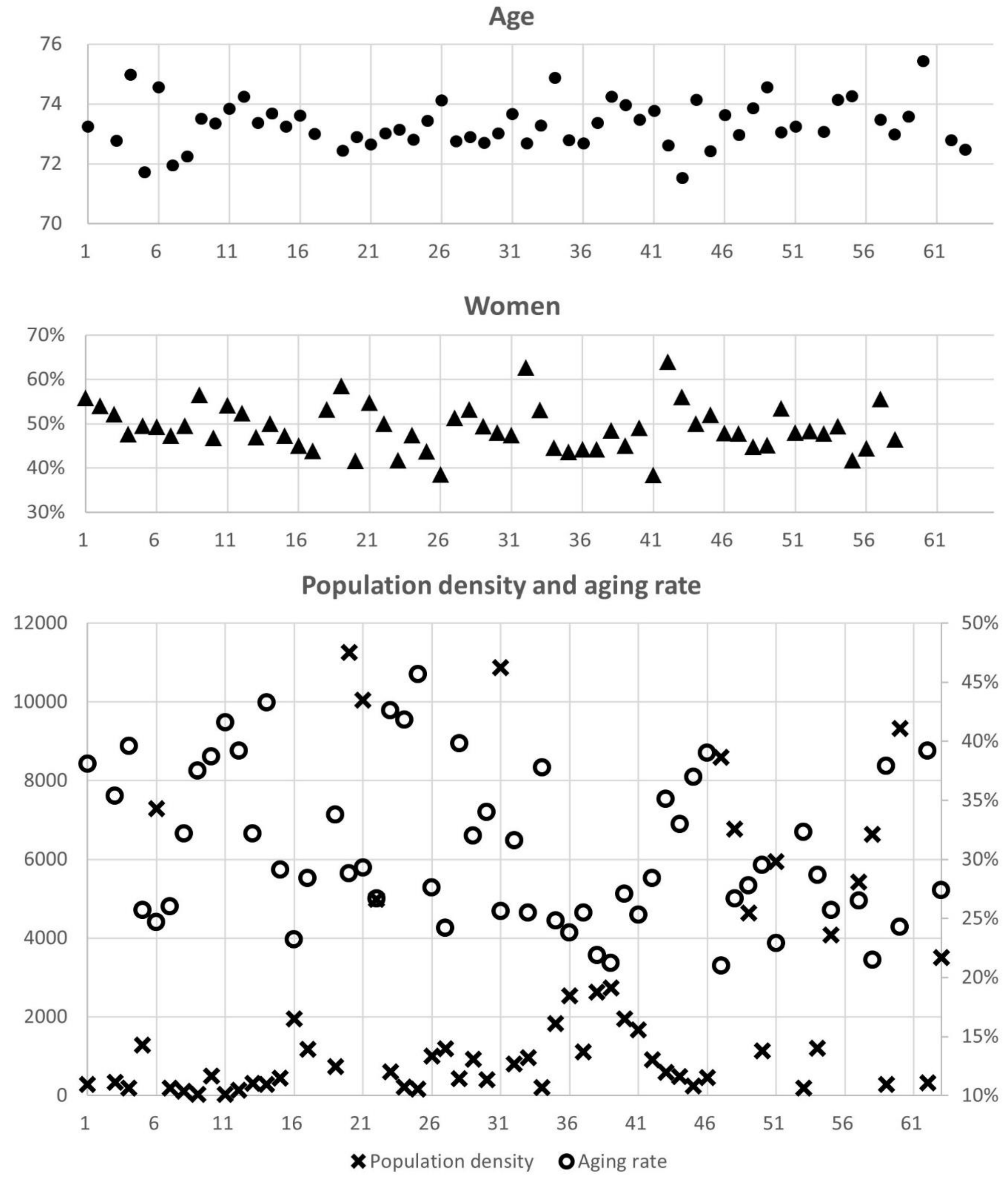

Supplementary Figure 2. Distribution of age, gender, population density, and aging rate. The X-axis indicates municipality IDs. The Y-axes indicate means of age (year), proportion of women (%), population density (people/km^2), and aging rate (%).

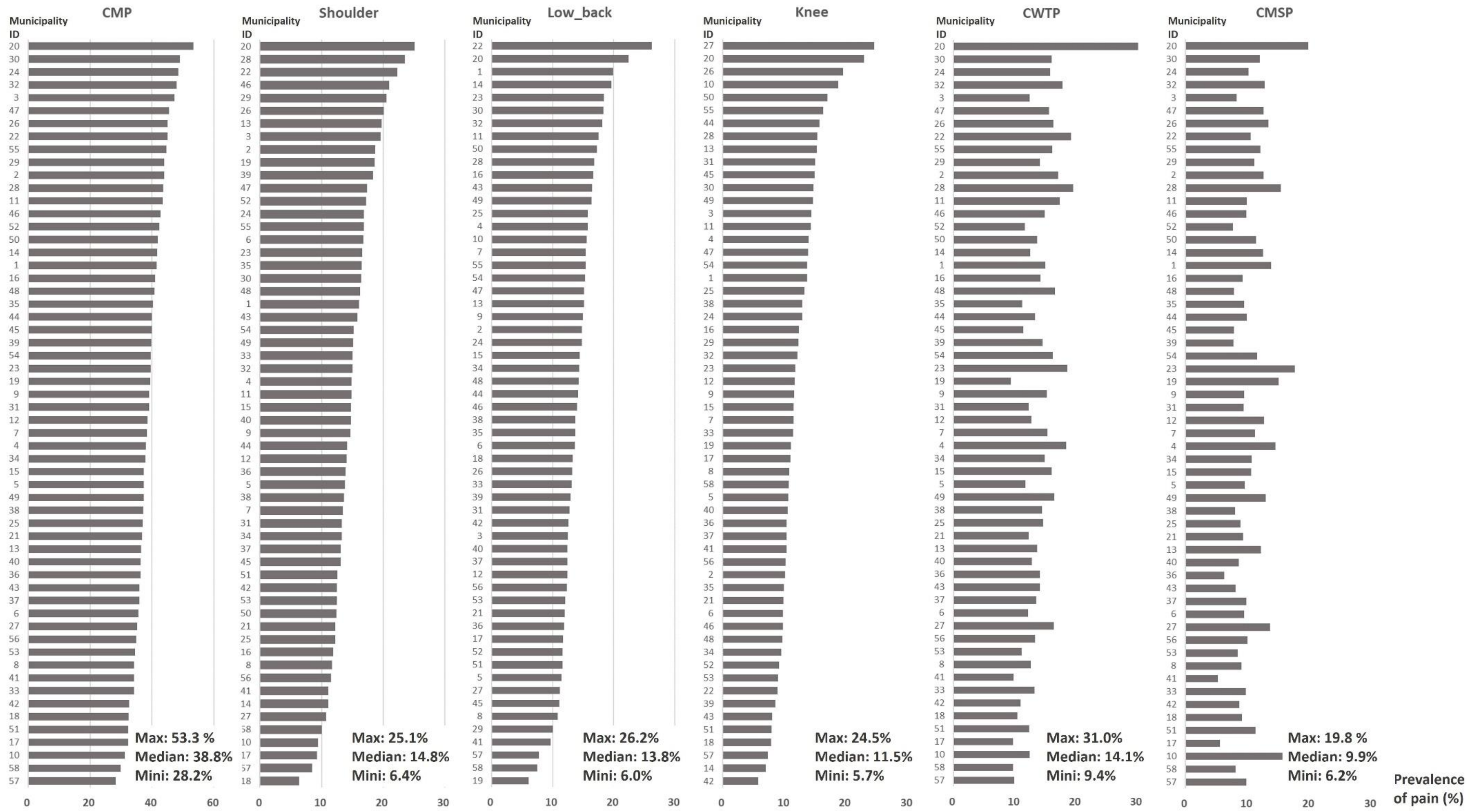

Supplementary Figure 3. Prevalence of each type of chronic pain. The X-axis indicates the prevalence of each type of chronic musculoskeletal pain (CMP) (%), and the Y-axis indicates municipality IDs. The maximum, median, and minimum prevalence of CMP are also indicated.
